# Delegation of implementation in project aid

**DOI:** 10.1007/s11558-020-09396-9

**Published:** 2020-11-21

**Authors:** Silvia Marchesi, Tania Masi

**Affiliations:** 1 Department of Economics, University of Milano Bicocca, Piazza dell’Ateneo Nuovo 1, Milano, I-20126 Italy; 2 Centro Studi Luca d’Agliano, Torino, Italy

**Keywords:** Foreign aid, World Bank projects, Delegation, Transparency, F35, O19, D83

## Abstract

In this paper we explore the factors that determine delegation of implementation in project aid. In particular, focusing on the importance of informational asymmetry between levels of government, we empirically assess whether this choice is influenced by the relative importance of the local information at the recipient country level. Moreover, we test whether this choice can in turn influence project performance. Using information on more than 5800 World Bank projects for the period 1995-2014, and controlling for characteristics at both country and project level, we find that transparency does influence the probability that a project is implemented locally rather than nationally. More specifically, a one standard deviation decline in transparency increases the probability of a locally implemented project by three percentage points. We also find that a local implementing agency may increase the probability of a successful project only up to a certain level of a country’s transparency.

**Electronic supplementary material:**

The online version of this article (10.1007/s11558-020-09396-9) contains supplementary material, which is available to authorized users.

## Introduction

In the last few decades, many developing countries have chosen to decentralize policymaking and implementation authority, particularly in the form of delegation of service delivery systems to local governments. The rationale behind such reforms lies in the efficiency argument, according to which local officials are better informed on local needs and are more capable of providing goods and services, promoting, thereby, efficiency and economic development (among other see Oates 1993; Bardhan 2002, 2016).^1^ Following this reasoning, the World Bank has been actively involved with decentralization policies in many developing countries, both funding projects aimed at building decentralized structures, and allocating loans to subnational governments.^2^

Although aid effectiveness could likely be improved by basing project designs on context-specific knowledge (e.g., Dixit 2009; Easterly 2008), the extent to which such information is actually used in aid implementation has rarely been investigated. An exception is provided by Dreher et al. (2017), who have shown that bilateral donors may choose to delegate some control rights over policies to recipients in order to exploit their local information.^3^

In this paper, we focus on the saliency of asymmetric information and the related importance of information transmission in projects implementation. In particular, we are interested in exploring the factors that might influence the choice of a central versus a local allocation of power in the implementation of project aid. Indeed, it seems that the choice of an implementing partnership is going to be one of the factors determining a project’ success (Shin et al. 2017). Nevertheless, little is still known about the choice of the implementation level. Our specific contribution is then to analyze which factors influence this choice in the case of World Bank projects, focusing particularly on the role of information.

Relying on a simplified version of the model developed in Dreher et al. (2018b), we show that decentralization will be chosen when the importance of the local knowledge is more relevant, which is going to be more frequent in less transparent countries (as we explain in Section 3). Therefore, we want to test, on the one end, whether a lack of transparency may influence, ceteris paribus, the donor’s choice in favor of a local implementing agency, and on the other, whether such decision can in turn affect the project performance.

Even though our analysis could be easily applied to other types of donors (Briggs 2019), in this paper, we have chosen to focus on the World Bank for two reasons. The first reason is the leading role of the World Bank as an international donor. The second reason is that the data available on the characteristics of World Bank projects are crucial for our empirical model (to our knowledge, no comparable publicly available data exist for other organizations).

Analyzing more than 5800 World Bank projects approved from 1995 to 2014, we find that the probability of a project being implemented locally, rather than nationally, declines as a country’s level of transparency increases. More specifically, a one standard deviation increase in transparency decreases the probability that a World Bank project will be implemented locally by three percentage points. We also find that a local implementing agency increases the likelihood of a successful project only up to a certain level of a country’s transparency. In particular, a locally implemented project, in a non-transparent country, has a predicted probability of success which is 64 percentage points higher than a nationally implemented one. On the other hand, the probability of success is 30 percentage points lower for a project which is locally implemented in a transparent environment.

Therefore, we argue that greater use of the local information, when most needed, is important to design better targeted interventions, since local realities might make policies that worked elsewhere not necessarily effective there. In this context, the Covid-19 crisis is going to increase even more the need of (local) good quality information, in order to be able to cope with the public health emergency.

The rest of this paper is organized as follows. Section 2 briefly summarizes the related literature, while Section 3 contains the stylized model. Section 4 describes the data. Section 5 describes the empirical model and our main results, while Section 6 presents the empirical results when testing for project performance. The final Section 7 concludes.

## Related literature

This paper relates to several strands of literature. The first is primarily concerned with the role of information in designing development reforms. Quite a few papers have argued that institutions and policies are context-specific and that conditional programs should suit better recipient countries’ specific needs, for their successful implementation (Asmus et al. 2017; Basurto et al. 2017; Besley and Persson 2011; Dreher et al. 2017; Hollyer et al. 2014; Honig 2018; Marchesi et al. 2011). Basurto et al. (2017) have shown that a decentralized allocation of subsidies in rural Malawi may offer informational advantages, despite being prone to elite capture. In a recent book, Honig (2018) argues that local information is particularly important to donors when they are working in fragile states (where levels of central government transparency are generally very low).

Building on the cheap talk literature (Crawford and Sobel 1982; Dessein 2002; Harris and Raviv 2005, 2008; Dreher 2017) examine the role of information transmission in the context of aid programs. They investigate the degree of leeway donors of foreign aid should grant to recipient governments when their preferences over how to implement the project are different, and both the donor and recipient possess some private information about the most effective policies. Their theoretical results show that donors should stay in control (centralized aid) of the use of their funds when their private information is more important than the private information of the recipient. When local knowledge is instead crucial, an increase in the difference of preferences between donors and recipients can increase the leeway that donors should grant the recipients (decentralized aid), as they become less likely to communicate truthfully. Testing the model using dyadic data, they find that misaligned interests and informational asymmetries indeed influence the shares of aid given as budget and project aid, which represent decentralized and centralized aid, respectively.

The second strand of literature to which this paper relates is the (vast) literature on decentralization and development topical both in economics and in political science (e.g., Asher and Novosad 2017; Asher et al. 2018; Bardhan and Mookherjee 2002, 2006, 2016; Dreher 2018a; Gadenne and Singhal 2014; Lessmann and Markwardt 2010, 2012, 2016; Oates 1993). Dreher et al. (2018b) explore the role of information transmission and misaligned interests across levels of governments in explaining variation in the degree of decentralization across countries. They theoretically show that the extent of misaligned interests and the relative importance of local and central government knowledge affect the optimal choice of policy-decision schemes. Their empirical analysis confirms that countries’ choices depend on the relative importance of private information and that the results differ significantly between unitary and federal countries.

Focusing on developing countries, Gadenne and Singhal (2014) consider how the trade-offs associated with fiscal federalism apply in this case and discuss reasons for their relatively low levels of decentralization. Lessmann and Markwardt (2010) find evidence that decentralization increases corruption in countries lacking bodies which can effectively monitor bureaucrats (such as a free press). More recently, Asher et al. (2018) focus on geographical distance, such as citizens’ physical remoteness from their administrators, as an important factor that constrains the state’s ability to provide public goods to all citizens.^4^ Using rich data on Indian villages, they find that reducing the distance between the state and its citizens does help to improve the efficiency of fiscal expenditure and to mitigate the large spatial disparities in living standards observed within many developing countries. While they focus on the costs to the state of supplying public goods and monitoring their quality, which increases with the distance between citizens and the state, we focus on the importance of the local knowledge for the optimal allocation of implementing power.

Indeed, despite the increasing number of aid projects allocated locally, the role of the federal structure of aid-receiving countries in affecting both aid allocation and efficiency has generally been neglected by the literature. An exception is provided by Lessmann and Markwardt (2012), who examine whether the degree of fiscal decentralization matters in explaining aid effectiveness. They find that foreign aid increases economic growth in highly centralized economies, while it may even be harmful in decentralized countries.^5^ In particular, coordination problems and higher corruption are found to be the more likely explanation for a reduction in aid effectiveness in decentralized economies. As they do, we also aim at creating a bridge between the literature on decentralization and that on aid effectiveness. Still, we depart from this study by focusing on the role of information and on delegation of implementation of projects -rather than country-level- aid.

Finally, this paper is related to a growing body of (empirical) literature which focuses on project-level aid, especially in the case of World Bank projects. See, for example, Denizer et al. (2013), Dreher et al. (2013), and Feeny and Vu (2017), Kilby (2000, 2001, 2013, 2015), Öhler and Nunnenkamp (2014), Shin et al. (2017). Denizer et al. (2013) find that only 20 percent of the total variation in project outcomes depends on a country’s institutions and macro-economic conditions, while the rest occurs (across projects) within countries. Bulman et al. (2017) compare World Bank projects with those of the Asian Development Bank, finding that country-level characteristics account for only 10-25 percent of the variation in project outcomes and discovering very few differences between these two institutions.

Relatively less attention has been devoted to the implementation phase. Kilby (2000), however, provides evidence on the importance of donor supervision in determining development project performance.^6^ More specifically, Kilby (2001) focuses on an agency problem between the World Bank and its borrower, due to misaligned objectives between the two. The monitoring component of World Bank supervision should then improve project performance by reducing the incentive to deviate. What is more, World Bank supervision-as-monitoring will have a greater impact when incentives to deviate are greater. Data from over 1,400 World Bank-funded projects support the importance of agency problems in determining performance and explaining the role of World Bank supervision. More recently, focusing on project characteristics, Kilby (2015) assesses the impact of World Bank project preparation on project outcomes, finding that, ceteris paribus, projects with longer preparation periods are significantly more likely to have satisfactory outcome ratings. Specifically, he finds substantially shorter project preparation periods for World Bank loans to countries that are geopolitically important (especially to the U.S.). This channel of donor influence provides a new angle to examine the cost of favoritism and the impact of project preparation. In a similar vein, Dreher et al. (2013), find that political motivations may hurt the performance of World Bank projects if a high-status country already faces economic difficulties.^7^

Annen and Knack (2015) theoretically demonstrate that bilateral donors delegate aid implementation to the multilateral agency to strengthen the policy selectivity of aid, incentivizing policy improvements in recipient countries, in turn improving aid’s development effectiveness. Their model shows that if one sufficiently large donor is policy selective in its aid allocations, there is no need for other donors to be policy selective. In particular, the International Development Administration (IDA) of the World Bank fits the assumptions and predictions of their model as it represents a dominant donor in most of its recipient countries and is much more policy and poverty selective than bilateral aid. More recently, Shin et al. (2017), find that the choice of an implementing partnership seems to be a significant indicator whether a World Bank development project will be successful or not. One of the important factors for a successful allocation would be the expertise of the related implementing partner, such as skills (knowledge and experience) and governance (organizational and institutional aspects).

Despite the importance of the implementing partner for project effectiveness (e.g., Shin et al. 2017), the factors that influence the implementing phase represent still an underexplored area of research. To the best of our knowledge, this is the first paper that investigates the determinants of a central versus a local allocation of implementing power in this context. In particular, we contribute to the literature analyzing the role of information in the delegation of implementation of World Bank projects.

## Stylized model

Following previous contributions (Marchesi et al. 2011; Dreher et al. 2017, 2018b), we focus on the saliency of asymmetric information and the related importance of information transmission in projects implementation. By adapting the theoretical model of Dreher et al. (2018b) to this framework, we identify the transmission of information between government levels with misaligned interests as an additional mechanism to understand the degree of decentralization in project implementation.

We rely on a simplified version of the model developed in Dreher et al. (2018b), which we modify in order to be applicable to the issues central to this paper.^8^ All detailed derivations and proofs are, however, shown in the original paper. We start outlining the baseline model, while the full model (which includes the World Bank) will be developed in the next sub-section.

In the current setting, the choice of a national vs local level of project implementation resembles the choice of a “decentralization vs centralization” policy scheme, and we plan to test whether informational asymmetry between central and local government and, more specifically, the importance of local knowledge, may explain (among other factors) the choice of a local implementing agency.

Asymmetry of information is assumed to be one-sided, namely, it is the local level of government which is assumed to have greater proximity to the ‘local business environment’ relative to central government officials, and to have better knowledge about the risks and opportunities of local investment projects. The private information of the agent is assumed to be soft, that is the agent cannot prove or certify her knowledge.^9^ What is more, the local government’s informational advantage may depend not only on how relevant its knowledge is per se, but also on how valuable such information is to the central government. For example, in highly intransparent environments, such informational advantage would be more salient compared to more transparent ones (which instead decrease the principal’s dependency on the local level).

### Baseline

The baseline model features two players, a central government and a local government, hereafter the principal and the agent, respectively. The central government must make a decision about the optimal level of implementation of a development project, denoted by *p*, and we assume that the agent has some informational advantage about the implementation of *p*. We define the optimal project *p*^∗^ as the number of project characteristics required to maximize the region’s welfare. The optimal project is defined by *p*^∗^ = *a*, where *a* is a stochastic variable that proxy for information observed only by the local government. *a* is uniformly distributed on the interval [0, *X*], the larger this interval, the larger the informational advantage of the local government.

The central government is assumed to be benevolent, hence its preferred project would correspond to the first best, that is: $p_{P}^{\ast }=p^{\ast }.$^10^ The central government, for simplicity (and analytical tractability), is supposed to maximize the following objective function:

1 $$ U^{P}={U_{0}^{P}}-(p-p^{\ast})^{2}, $$

where *U*^*P*^ decreases with the distance between the actually implemented project *p* and the optimal project *p*^∗^, and ${U_{0}^{P}}=U^{P}(p^{\ast }).$^11^

The local government maximizes:

2 $$ U^{A}={U_{0}^{A}}-(p-p_{A}^{\ast})^{2}, $$

which is decreasing in the distance between the implemented project *p*, and the recipient government’s preferred project $p_{A}^{\ast }$, with $U_{0} ^{A}=U^{A}(p_{A}^{\ast })$.^12^ The optimal project choice of the local government deviates from the optimal project *p*^∗^ by a factor *b* > 0 (i.e., $p_{A}^{\ast }=p^{\ast }-b)$. The local government cares about the region’s welfare, but is also assumed more subject than the national apparatus to the pressure of some (local) interest groups benefitting from structural distortions (e.g., Drazen 2002).^13^*b* captures the extent to which the project choice of the local government may deviate from its optimal level *p*^∗^, due to the pressures of interest groups opposing the project.

Therefore, the difference in optimal policies is given by

3 $$ p_{P}^{\ast}-p_{A}^{\ast}=p^{\ast}-(p^{\ast}-b)=b, $$

where *b* reflects the extent of agency bias, which proxies for all factors that might lead to a deviation of the local government’s preferences from *p*^∗^, due to the pressure of local interest groups and re-election concerns (among others). We assume that the misalignment of interests between the principal and the agent exclusively depend on the agent’s bias *b*.

Whenever the interests of the two levels of government differ, the quality of the information will depend on such conflicts of interest, with the central government rationally expecting the information transmitted by the local one to be distorted (*cheap talk game,* Crawford and Sobel 1982). Within this broad perspective, this paper focuses on the comparison of two types of incentive structures, relative to the quality of the transmitted information: “centralization” and “decentralization.” Under centralization, project implementation is assigned to the national government, whereas under decentralization, it is assigned locally.

**Fig. 1:**
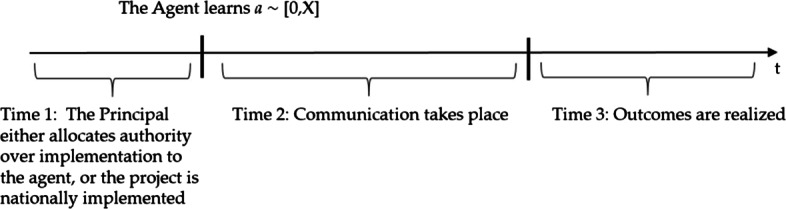
Timeline

As principal, the central government can choose between centralization or decentralization. As Fig. 1 shows, events unfold in three stages: allocation of control rights by the principal, communication, and project implementation. In the first stage, the principal either allocates authority over implementation to the agent or implements the project nationally. After the first stage of the game, the real state of the world is revealed to the agent. Then, in the second stage, communication takes place. Under centralization, the local government sends a ‘message’ to the central one regarding its ‘local knowledge’. Upon receiving the message, the central government updates its beliefs and implements the project. Under decentralization, the local government implements the project. Finally, in the third stage, outcomes are realized. Since information transmission is untruthful, the principal either delegates control or takes an uninformed action.

As shown by Dreher et al. (2018b), by opting for centralization, the principal minimizes the costs of misaligned incentives at the cost of the under-utilization of the agent’s private information. Under delegation, the agent’s private knowledge is fully exploited, but the results can deviate from the principal’s optimal policy (loss of control). Hence, the principal prefers delegation to centralization unless the agency bias is so big that informative communication would not be feasible. In contrast, keeping the agency bias constant, centralization dominates delegation if the uncertainty about the environment is sufficiently small.

### The model with the World Bank

In this section, we analyze what happens in the case in which the model features three actors: the World Bank (which is now the principal), the central government and the local government.^14^ The World Bank is assumed to have no informational advantage itself. Moreover, it is assumed to be a benevolent multilateral institution, helping countries implement projects to raise the quality of people’s lives. Hence, its preferred project would correspond to the first best, that is: $p_{W}^{\ast }=p^{\ast }.$ The Word Bank then maximizes the following objective function:

4 $$ U^{W}={U_{0}^{W}}-(p-p^{\ast})^{2}, $$

where *U*^*W*^ decreases with the distance between the actually implemented project *p* and the optimal project *p*^∗^, and *U*0*W* = *U*^*W*^(*p*^∗^). As we assume a benevolent institution, we do not consider the World Bank’s concern for the interests of some “special” shareholders, nor we consider the importance of ideology in reforms’ design, which was also found to matter.^15^ For example, Smets et al. (2013) investigate how government ideology matters for the success of World Bank loans, finding that World Bank staff will invest more effort in designing an economic policy loan when faced with a left-wing government. These are indeed strong assumptions but allow us to focus on the issue of information transmission and its implications for the choice of the implementation level.^16^

We now need to consider two alternatives, either the World Bank delegates or keeps to itself decision making over the choice of the implementation. Let’s consider, first, the possibility that the World Bank delegates decision making over the choice of the implementation level to the national government.^17^ In this case, as previously demonstrated, delegation to a local implementing agency should prevail when the advantages of the local information are high enough to dominate the costs due to loss of control, but not for other reasons.

Finally, we consider the case in which the World Bank autonomously decides whether to delegate authority to the national or to the local government. Furthermore, for simplicity of exposition, we also assume that the donor can always take (and enforce) the decision regarding the level of implementation.^18^ Since the World Bank is not assumed to have any informational advantage per se, then it will be facing a trade-off between loss of control due to the conflict of interest (when choosing a nationally implemented project), and loss of information due to only a partial utilization of the local knowledge (when choosing a locally implemented project). In particular, in this setting, we exclude the possibility that the donor delegates control to the local government if it is less trusting of central government institutions, that is unrelatedly to the donor’s need for local information in non-transparent settings. As previously demonstrated, it would opt for delegation when, for a given bias, the uncertainty about the environment is sufficiently big.

### Empirical implication

The model provides some testable implications that can be derived from the theory. The main prediction of the model is that delegation should prevail if, ceteris paribus (that is controlling for project and country characteristics, including a proxy for the agency bias), the importance of the local knowledge is sufficiently big, which is going to be the case in more opaque countries. In fact, in countries that lack information transparency, for the national authorities it would be more difficult to obtain information from sub-national government institutions, and the need to delegate authority to a local implementing agency should increase.^19^

More specifically, we argue that the availability of information that is recorded can be limited in developing countries. This decreases the share of “hard” information that can easily be transferred and increases the importance of private “soft” knowledge.^20^ The relative share of hard to soft information, in turn, may depend on a country’s transparency. In fact, less transparency may make the existing informational asymmetry more salient and lead the principal to delegate control rights over policy implementation (locally implemented project). Therefore, we argue that the less transparent a country is, the more critical the local information will be. In more opaque countries, information would not be publicly available at the central level, increasing, ceteris paribus, the government’s dependency on the local level, with less information being available in cases where no ”decentralization” is chosen.^21^

Finally, due to the trade-off between loss of control and loss of information, the choice of the implementing agency may have an impact on the project performance. In particular, we argue that, if the implementation power is allocated locally when a country is transparent and hence the informational asymmetry is not salient, the costs due to loss of control may overcome the informational advantages, decreasing aid effectiveness.

In sum, we plan to test the two following hypotheses:

*i. The probability of a local implementing agency decreases as country’s transparency increases.*

*ii. The probability of success of locally implemented projects decreases as country’s transparency increases.*

## Data

We use the AidData (2016) dataset, which includes 5881 World Bank projects in the International Bank for Reconstruction and Development (IBRD) and International Development Association (IDA) lending lines, approved from 1995 to 2014. Among other project characteristics, AidData provides information on the body which is responsible for implementing the project without explicitly coding them. We decided to consider five main types of implementing agency: national and local government, public and private company, and non-governmental organization (NGO).

Then, we classify an agency as national (local) when the government responsible for project implementation is the national (local) one, and we code as a public (private) company an agency which is owned or regulated by the government (the private sector). When this information was missing, we collected the required data through the World Bank’s project-specific documentation.

Following this procedure, we have to exclude 30 projects due to data availability constraint. Furthermore, since we are interested in the determinants of national vs local allocation of power, we exclude projects implemented by supranational agencies in more than one country (115 projects). On the other hand, when projects are implemented simultaneously by several agencies, we attribute the same project to each of the involved agency. Our sample includes, thereby, 5736 projects that are implemented in 143 countries.

**Fig. 2:**
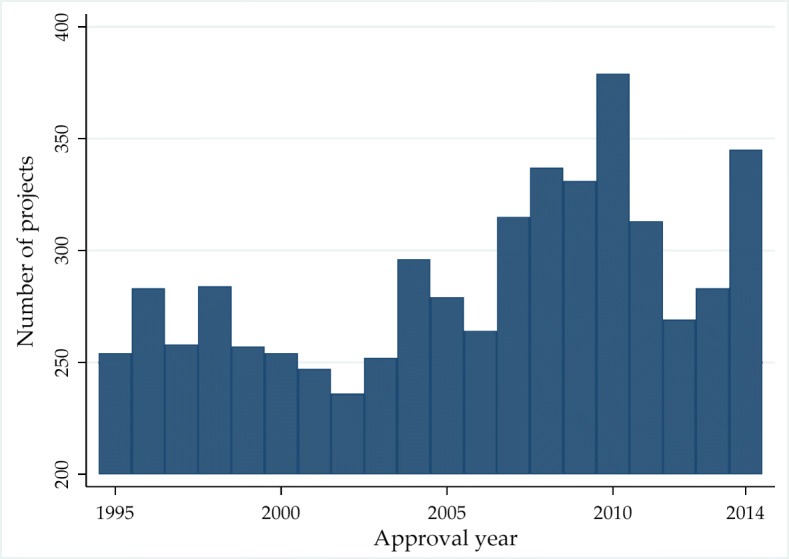
Project distribution over time

**Fig. 3:**
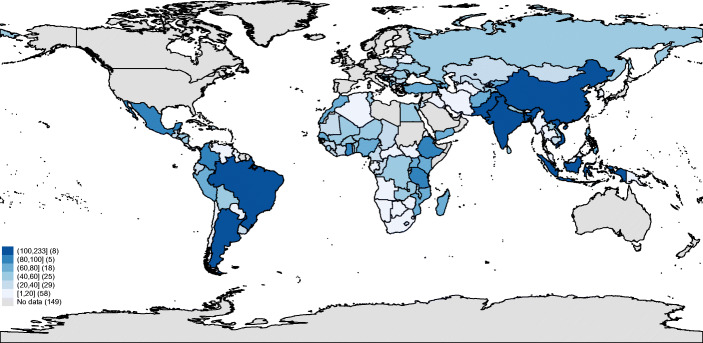
Project distribution across countries

Figure 2 shows the evolution of World Bank projects over the sample period. We observe that the number of projects per year ranges from 250 to 300 until the year 2007 (when it exceeded 300), and it then reaches its peak in 2010 (when 379 projects were approved). After a substantial decline in 2012 and 2013, in the last year of the sample, we detect a sharp increase in the number of projects again. Then, Fig. 3 shows the worldwide distribution of World Bank projects. As we can see, Pakistan, Bangladesh, Indonesia, Vietnam, India, China, Brazil, and Argentina obtained more than 100 projects during the sample period (with a maximum of 233 projects in China). On the other hand, a large number of countries were involved in less than 20 projects during the same period. Table A1 in the Online Appendix lists all countries included in the sample and report the corresponding number of projects.

Table 1 shows the distribution of World Bank projects by our five types of implementing agency. As we can see, the vast majority of the projects is implemented by national governments, typically by the ministries who are directly responsible for the project’s sector. On the other hand, there are 767 projects which are implemented by subnational governments. Furthermore, 374 and 22 projects are implemented by public and private companies, respectively. Finally, only 11 projects are carried out by NGOs.

**Table 1:** Project distribution across implementing agencies

Implementing agency	Number of projects
National government	4689
Local government	767
Public company	374
Private company	22
Non-Governmental Organization	11

Notes: Authors’ calculations based on AidData (2016)

Then, Table 2 presents the distribution of the World Bank projects in our sample, according to our final classification between national and local implementing agency. Public companies are coded as national (local) when the level of the government owning or regulating them is national (local). We need to exclude 11 projects which are exclusively implemented by either private companies or NGOs since, in both cases, it is impossible to attribute them to any government level.^22^ For the same reason, we exclude 59 projects involving both local and national agencies simultaneously. Consequently, the resulting dataset includes 5666 projects widely distributed across regions and sectors, as shown in Table 3 and Table 4.

**Table 2:** Project distribution: national vs. local implementing

		Local implementing agency		
		0	1	Total
National implementing	0	11	741	752
agency	1	4,925	59	4,984
	Total	4,936	800	5,736

Notes: Authors’ calculations based on AidData (2016)

**Table 3:** Project distribution across regions

	Total		Local implementing agency		
				%	%
	Number	%	Number	(local impl. agency)	(total projects)
South Asia	644	11.4	204	27.5	3.6
Europe and Central Asia	1103	19.5	83	11.2	1.5
Middle East and North Africa	354	6.2	14	1.9	0.2
Africa	1633	28.8	61	8.2	1.1
Latin America and Caribbean	1107	19.5	166	22.4	2.9
East Asia and Pacific	825	14.6	213	28.7	3.8
Total	5666	100.0	741	100.0	13.1

Notes: Authors’ calculations based on AidData (2016)

**Table 4:** Project distribution across sectors Total

	Total		Local implementing agency		
				%	%
	Number	%	Number	(local impl.	(total
				agency)	projects)
Agriculture, fishing, and forestry	521	9.2	110	14.8	1.9
Education	512	9.0	37	5.0	0.7
Energy and mining	459	8.1	58	7.8	1.0
Finance	294	5.2	6	0.8	0.1
Health and other social services	786	13.9	54	7.3	1.0
Industry and trade	314	5.5	30	4.0	0.5
Information and communications	52	0.9	0	0.0	0.0
Public Administration,	1516	26.8	89	12.0	1.6
Law, and Justice					
Transportation	677	11.9	181	24.4	3.2
Water, sanitation and	535	9.4	176	23.8	3.1
flood protection					
Total	5666	100.0	741	100.0	13.1

Notes: Authors’ calculations based on AidData (2016)

*Africa* absorbs the largest proportion of World Bank projects, even if local agencies implement only 61 of them. Conversely, in both *Asia* and *Latin America* there is the highest proportion of “local projects.”^23^ Furthermore, Table 4 shows the distribution across the ten major project sectors, as classified by the World Bank.^24^ First of all, we observe that only 13 percent of all the projects in our sample are locally implemented. Then, while the largest single sector is *Public Administration*, *Law*, *and* *Justice* sector, this sector accounts for only 12 percent of all locally implemented projects. Implementation power is instead more likely to be decentralized when the project falls into the *Transportation* or *Water, Sanitation and Flood Protection* sectors. Finally, while at least 52 projects belong to the *Information and Communications* sector, none of them is implemented by local agencies.

**Fig. 4:**
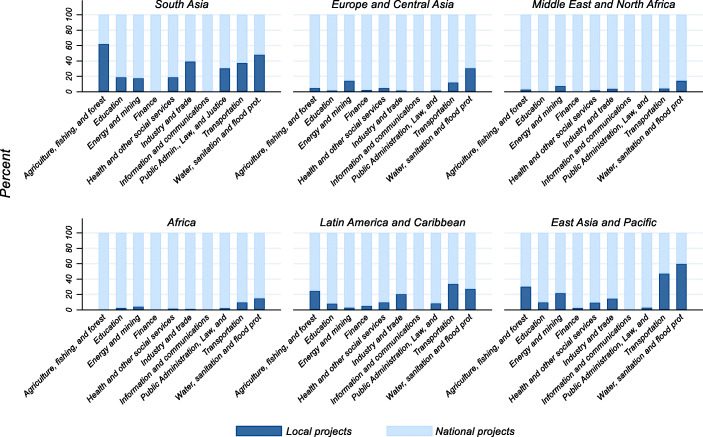
Project distribution across regions and sectors (percentage of local over total projects)

Figure 4 confirms that *Africa* and the *Middle East and North Africa* have the lowest percentage of local projects across each sector. On the contrary, the rate of local projects in *Asia* is 20%, or higher, in all sectors but *Finance* and *Information and Communications.* In that region, 60% of the projects in *Agriculture, fishing and forest* are implemented by local partners, while, in *East* *Asia and Pacific,* the same percentage is reached in the sector *Water, Sanitation and Flood Protection.*

### Project evaluation

We use the World Bank’s Independent Evaluation Group (IEG) rating, reported in the AidData (2016) dataset, to test for the role of a country’s transparency in project performance, under delegation of implementation.^25^

The IEG is an independent unit within the World Bank Group that reports directly to the Board of Executive Directors. The World Bank prepares the Implementation Completion Reports (ICRs) by assigning ratings for all Board-approved projects. The IEG is responsible for reviewing all the ICRs, assessing programs and activities, making recommendations, and disseminating lessons learned from experience. A subset of about 25% of all completed projects is subject to an in-depth field-based evaluation, called a Project Performance Assessment Report (PPAR).

ICR ratings are less likely to have a systematic (upward) bias because, on the one hand, there is the possibility that IEG will audit the project, and, on the other, project outcomes have little impact of staff careers (Dreher et al. 2013). In turn, both circumstances depend on the fact that (i) the World Bank focuses on making new loans (rather than on how old loans turn out); (ii) frequent staff rotation makes attributing credit or blame difficult.

In this paper, we consider the *Outcome Rating*, which is a composite indicator based on the relevance of design, efficacy and efficiency of the projects (see IEG 2018), and can take the following six evaluations: *Highly Unsatisfactory, Unsatisfactory, Moderately Unsatisfactory, Moderately Satisfactory, Satisfactory, Highly Satisfactory*. Following the bulk of the literature, we use a binary indicator, *Satisfactory*, which is equal to 1 if the project is evaluated at least *Moderately Satisfactory*, and 0 otherwise.

### Variable of interest

We argue that the share of “hard” to “soft” information is increasing with a country’s transparency, especially in developing countries. Hence, the local (soft) knowledge would be more important the less transparent a country is. If transparency is generally higher, more information is publicly available at both the central and local level. It thus decreases the principal’s dependency on the local information, with more information being available in cases where no delegation is chosen. In our empirical analysis, we use four different proxies for a country’s transparency.

The first one, the *Share of No Missing Data* (*Transparency*), is an indicator of information transparency given by the share of the 1260 data series included in the World Development Indicators (World Bank 2013) for which data are available for a given country and year (Dreher et al. 2017).^26^ Then, we use the *Share of No Missing Economic Data* constructed by Hollyer et al. (2014), which focuses on standard economic indicators (relating to economic policy and debt) as indicators of transparency.^27^

The third proxy is the Combined Transparency Index constructed by Williams (2015), which is a measure for the general data transparency in a country. Williams (2015) uses a relatively new methodology, similar to Transparency International’s Corruption Perceptions Index, to construct composite indicators of what they call Informational Transparency, and Accountability. These new indicators use data from 29 sources, with scores being derived annually between 1980 and 2010 across more than 190 countries. We use the combined index of political and information transparency, where lower values indicate a lower ability to get access to reliable information.

Finally, we use an indicator measuring the degree of Press Freedom (taken from Freedom House 2011, on a scale from 0-100). As widely recognized in the literature, a free press can make politicians and bureaucrats more accountable, applying constraints upon their actions and raising the opportunity cost of engaging in corrupt or unethical behavior (Besley and Burgess 2001, 2006; Brunetti and Weder 2003). Freedom House assesses the degree of print, broadcast, and digital media freedom. It categorizes each country with a score that determines the status designation as free, partly free and not free.^28^ However it is measured, higher transparency is associated with lower importance of the local knowledge.

### Control variables

Following Denizer et al. (2013), we consider both project-level and country-level control variables. Among the first group of control variables, we include *Total Amount*, that is the amount of commitment measured in million U.S. dollars, to capture project complexity. We also include *Investment Project*, that is a dummy equal to 1 if a specific investment project is financed, and equal to 0 if the project consists instead in development policy lending (i.e., capturing general budget support), and a dummy indicator for *IBRD* projects as opposed to IDA lending. Nearly 80 percent of the projects in our sample consist of investment projects, confirming the low and declining importance of budget support, and *IBRD* projects account for 35 percent of total projects.^29^ Finally, we control for the project sector, following the same classification presented in Table 4. Including the project sector is fundamental, as it allows us to control for the projects that cannot be “arbitrarily” allocated to the central or to the local government but which need to be implemented either locally or nationally because of their intrinsic characteristics.^30^

As country-level variables, we include the *Ethnic Fractionalization* index taken from Alesina et al. (2003) to capture the influence of local interest groups, as in previous studies (e.g., Dreher et al. 2017 and 2018a). This index is widely used in empirical studies and is available for a large number of countries. We expect that greater diversity of the population will imply, on average, more substantial differences in the policy preferences between the central and the local government. Therefore, the greater the fractionalization, the greater the distance between the two levels of government. We also control for*Internal* and *External Conflicts,* which in turn may exacerbate such bias. Data comes from the International Country Risk Guide (PRS Group 2012).

What is more, we consider the (cumulative) number of World Bank past locally implemented projects, received by each recipient country up to the project year. This variable allows us to take into account whether the previous use of local implementing partners may have some permanent effect, which in turn may influence the current choice of an implementing agency.

We include *Bureaucratic Quality* from the International Country Risk Guide (PRS Group 2012), in which higher scores indicate that the bureaucracy has the strength and expertise to govern. As argued by Knack and Rahman (2007), skilled government bureaucrats provide important public goods that, among other effects, improve the development impact of all aid projects in the country. We expect that the higher the quality of the bureaucracy at the national level, the higher the probability that local governments’ bureaucrats are as qualified as those in national governments (Lessmann and Markwardt 2016). Thus, the incentives to delegate the project to a local implementing agency is increasing with bureaucratic quality. Moreover, we take into account whether the country has a unitary or federal structure (*Federal System*) using data available from Norris (2008), since the probability of delegation should be higher in federal than in the unitary country.^31^

We also control for *GDP per capita* to consider the level of development, and *Population*, which also captures “need,” but can be taken as proxy for the ease of obtaining a country’s political cooperation as well, since smaller countries are easier to “buy” (e.g., Boone 1996). This choice is also consistent with the standard specification in the decentralization literature, according to which bigger and richer countries are more likely to be decentralized.^32^ Finally, we also include regional dummies and time fixed effects to account for unobserved characteristics that might be correlated with our variables of interest.

Table A2, in the Online Appendix, contains definitions and sources, Table A3 provides descriptive statistics, and Table A4 shows the correlations of all variables included in the analysis.

## Method and results

In this section, we examine the determinants of decentralized implementing agencies using logit and multilevel logit models to estimate the following equation:

5 $$ l_{i,k,j,t}=\upbeta T_{j,t-1}+\gamma X_{i,t}+\delta Z_{j,t-1}+\eta_{k}+\mu_{j}+\tau_{t}+u_{i,k,j,t} $$

where *l* is a binary indicator of project *i*, in sector *k*, in country *j*, at time *t*, which is equal to one if the project is implemented by a local implementing agency. *T* is the information transparency indicator evaluated at time *t* − 1, *X* denotes the set of control variables related to project *i* at time *t*, while *Z* includes country-level variables evaluated at time *t* − 1 and *η*_*k*_ are sector dummies. Finally, *μ*_*j*_ and *τ*_*t*_ are region- and year-fixed effects, respectively and *u*_*i*, *k*, *j*, *t*_ is the error term. We include regional dummies rather than country fixed effects because we want to use cross-sectional variation for identification in addition to within-country variation. In particular, when we include country fixed effects, more than seventy countries are dropped from the analysis, due to the limited variation in the dependent variable.^33^

The results from the logistic regression may be biased due to the violation of the observation independence assumption. Thus, we take into account the way in which World Bank projects are nested into clusters, and we implement a multilevel logistic regression. Multilevel models seem particularly appropriate for research designs in which data are organized at more than one level (nested data). In particular, the units of analysis are projects, at a lower level, which are nested within aggregate units, that is countries, at a higher level.^34^

The results are presented in Table 5. Columns 1-4 show the results from the logistic regression while columns 5-7 present the results of the multilevel logit. The results of column 1, which only includes project-level variables, show that the coefficient of *Transparency* is negative and significant at the one percent level, meaning that greater *Transparency* is negative associated to the probability of having a local implementing agency. As for the control variables, the coefficient of the dummy for *IBRD* projects is positive and significant at the one percent level, as expected, while the coefficients of both the committed amount and the investment dummy are not significant at conventional levels.

**Table 5:** Decentralization of implementing agencies, Logit and Multilevel Logit

	(1)	(2)	(3)	(4)	(5)	(6)
VARIABLES	Logit	Logit	Logit	ML	National sectors	Local sectors
Transparency	− 2.013***	− 1.466***	− 3.779**	− 4.343***	− 8.497***	− 3.732***
	(0.351)	(0.519)	(1.932)	(0.664)	(1.384)	(0.778)
Total amount	0.000	− 0.000	− 0.002***	− 0.002***	− 0.002***	− 0.002***
	(0.000)	(0.000)	(0.001)	(0.000)	(0.001)	(0.001)
Investment projects	− 0.010	0.088	− 0.063	− 0.177	− 0.526*	− 0.024
	(0.136)	(0.187)	(0.235)	(0.194)	(0.278)	(0.316)
IBRD	0.992***	0.763***	0.081	0.614*	0.524	0.725**
	(0.125)	(0.163)	(0.337)	(0.371)	(0.680)	(0.359)
Ethnic fractionalization		− 0.024***	0.001			
		(0.003)	(0.005)			
Federal system			0.880***			
			(0.267)			
Past local projects			0.035***			
			(0.004)			
Bureaucratic Quality			0.504***			
			(0.184)			
GDP per capita (log)			− 0.724***			
			(0.211)			
Population (log)			0.224**			
			(0.092)			
Internal conflict			0.091			
			(0.062)			
External conflict			− 0.009			
			(0.077)			
Observations	4,997	2,857	2,421	4,997	2,368	2,629
Sector dummies	YES	YES	YES	YES	YES	YES
Regional dummies	YES	YES	YES	YES	YES	YES
Year FE	YES	YES	YES	YES	YES	YES
Number of groups				139	133	135

Notes: Transparency is Share of No Missing Data. Robust standard errors are reported in parentheses. *** p < 0.01, ** p < 0.05, * p < 0.1

The coefficient of *Transparency* remains significant but slightly decreases in size in column 2, in which we also control for *Ethnic Fractionalization*. The sign of the coefficient of this variable indicates that, as the racial and linguistic heterogeneity increases, the distance between the preferences of the World Bank and that of the recipient governments also increases, leading to lower incentives to delegate the project implementation to a local agency (the agency bias increases).

After including *Ethnic Fractionalization*, however, more than 2000 observations are lost due to a lack of data. Hence, in order to take more seriously the problem of missing data, we have extended the number of observations by using an updated version of our dataset (columns 1 and 2 of Table A7, in the Online Appendix) and implementing a multiple imputation procedure (columns 3 and 4 of Table A7). In the updated version of our dataset, we use, as a proxy for *Ethnic Fractionalization*, the Historical Index of Ethnic Fractionalization Dataset (Drazanova 2019), which is time varying.^35^ In both cases, the negative and highly significant relation between Transparency and local implementation is confirmed.^36^

In column 3 of Table 5, we include all country-level variables. In this case, the coefficient of our variable of interest is still negative and significant. As for the control variables, the amount of the project is negatively correlated, although its coefficient is rather small. Looking at the country characteristics, we find that *Past local projects* is positive and significant, meaning that the previous use of local implementing partners is associated with a further use of them. The *Bureaucratic Quality* does play a positive role in the choice of the implementation level, as the *Population*, confirming that the more populated a country is, the higher the incentives to delegate to a lower level of government, as the literature on decentralization suggests. Moreover, the coefficient of *Federal system* is positive and significant, showing that federal countries are indeed more likely to have a local level of implementation of a World Bank project, as the intuition would suggest.*GDP per capita* has a negative and significant coefficient, which instead goes against a standard result of the decentralization literature, but could be explained by the fact that poorer countries are just more in need of World Bank intervention (independently of the level of project implementation). *Ethnic Fractionalization, and Internal* and *External Conflict* are not significant instead.

In column 4, we estimate the multilevel logistic model. A likelihood-ratio test comparing this model to the ordinary logistic regression is significantly in favor of the former specification. Thus, we consider this specification as our preferred one.^37^ The coefficient of *Transparency* is still negative and highly significant. Considering its marginal effects, one standard deviation increase in transparency would decrease the probability of having a local implementing agency by about three percentage points. Although a 3 percentage point change may not seem large, the sample average is only 13 percent so a 3 percentage point change is large in relative terms.

Finally, columns 5 and 6 present the results of two alternative specifications, in which we split our sample distinguishing by project sectors. More specifically, we compare the relative influence of *Transparency* by considering projects that belong to national and local level in two separate regressions. We consider *Education*, *Finance, Industry and Trade*, and *Public Administration, Law, and Justice* as *national* sectors, while we classify *Energy and mining*, *Health and other social services*, *Information and communications*, *Transportation*, *Water, sanitation and flood protection* as *local*. In particular, we expect that the choice between delegating project implementation at the local level vs keeping it at the national one, should more likely be made for projects belonging to the first group rather than to the second one. In other words, in the case of projects classified as *local*, there is not a real trade-off as the local knowledge is probably too important for actually choosing to implement them nationally. Thus, the projects for which a real choice can be made (the so called “switchers”) are more likely to belong to the first group.

The results of columns 5 and 6 show that the coefficient of *Transparency* is negative and significant in both specifications, but the size of the coefficient is much bigger, in absolute terms, for projects that fall into the *national* sectors (column 5), than those which belongs to the *local* one (column 6). As can be seen, in column 6, the size of the coefficient of *Transparency* is comparable to that of the full sample. Therefore, the role of information, in the choice of the delegation of implementation, seems more important in the case of the “switchers” than in the full sample, as expected.

### Robustness checks

This section contains a number of robustness checks. We start by replicating the estimates presented in Table 5 using alternative indicators of a country’s transparency. Then, we replicate the estimates of Table 5 by restricting the sample to projects evaluated either as “satisfactory” or “highly satisfactory” and only to countries with “good political institutions.” We estimate a multilevel logistic model using each of these alternative measures for transparency and controlling for project-level covariates. As can be seen in Table 6, information transparency is negatively and significantly related to the probability that a local agency implements a World Bank project in all specifications. As for the control variables, *Local projects* are still positive and significant, while the coefficient of *Total amount* is always negative but small.

**Table 6:** Robustness checks

	(1)	(2)	(3)	(4)	(5)
	Share of No missing Economic Data	Press Freedom	Combined Transparency Index	Satisfactory projects	Good Institutions
Variables	ML	ML	ML	ML	ML
Transparency	− 2.441***	− 0.805***	− 0.052***	− 3.992***	− 4.431***
	(0.342)	(0.128)	(0.008)	(0.884)	(0.887)
Total amount	− 0.002***	− 0.002***	− 0.002***	− 0.002***	− 0.002***
	(0.000)	(0.000)	(0.001)	(0.001)	(0.001)
Investment projects	− 0.231	− 0.353*	− 0.166	− 0.558**	− 0.206
	(0.197)	(0.181)	(0.212)	(0.283)	(0.244)
IBRD	0.042	0.224	0.826**	0.469	0.662
	(0.335)	(0.354)	(0.381)	(0.432)	(0.463)
Observations	4,739	4,739	4,312	2,437	2,789
Number of groups	138	137	135	130	95
Year FE	YES	YES	YES	YES	YES
Sector dummies	YES	YES	YES	YES	YES

Robust standard errors are reported in parentheses. *** p < 0.01, ** p < 0.05, * p < 0.1

Going into more detail, columns 1-3 in Table 6 present the results obtained using three alternative indicators of information transparency. As described in Section 4.2, we use the transparency index provided by 2014, the in dicator of press freedom provided by Freedom House (2012), and the Transparency index built by Williams (2015). Considering one standard deviation increase in each transparency indicator, the magnitude of their impact is comparable to the one obtained in the baseline specification.

Columns 4 and 5 present the results obtained by restricting the sample, first, to projects that are evaluated at least as *Moderately satisfactory* (column 4), then to projects implemented by democratic regimes, which are countries that achieved a score equal to or greater than 6 in the polity2 indicator (columns 5). In this way, we focus on projects which are less likely to be influenced by political aspects. Since geopolitical factors have generally been found to negatively affect projects’ performance (e.g., Dreher et al. 2013 and Kilby 2015), projects obtaining a good rating should, on average, be more likely to be independent from political factors. In a similar vein, in countries with good political institutions, the allocation of the implementing power should less likely depend on the pressure of interest groups.

In both cases, the transparency indicator is the *Share of No Missing Data.* In column 4, a one standard deviation increase in *Transparency* would decrease the probability of a local implementing agency by about two percentage points, while, in column 5, a one standard deviation increase in *Transparency* would decrease the probability of a local implementing agency by about three percentage points.

## Information transparency and project performance

In this section, we want to assess whether informational asymmetries may also affect a project’s performance, under delegation of implementation. Following Dreher et al. (2013), we estimate the following logit and conditional logit models (with standard errors clustered at the country level):

6 $$ \begin{array}{@{}rcl@{}} p_{i,k,j,t}&=&\upbeta T_{j,t_{app}-1}+\gamma l_{i,k,j,t_{app}}+\delta T_{j,t_{app}-1}\ast l_{i,k,j,t_{app}}+\theta X_{i,t_{app}}+\lambda Z_{j,t_{app}-1}\\ &&+\eta_{k}+\tau_{t_{app}}+\varepsilon_{i,k,j,t_{app}} \end{array} $$

where *p* is a binary indicator, which is equal to one if project *i* is evaluated at least as Moderately Satisfactory, in country *j*,at time *t*. *T* is the information transparency indicator one year before the projectapproval, at time *t*_*app*_ − 1, and *l* indicates whether the project was implemented by a local implementing agency at the time of project approval. *X* denotes the set of control variables related to project *i* at time *t*_*app*_,while *Z* includes country-level variables evaluated at time *t*_*app*_ − 1. Finally, *η*_*k*_ are sector dummies and $\tau _{t_{app}}$ denote year fixed effects (to control for common trends) and $\varepsilon _{i,k,j,t_{app}}$ is the error term. We also include regional dummies or country fixed effects, according to the chosen specification.

Most importantly, we interact the two variables *T* and *l*, in order to test whether the performance of a locally implemented project may depend on the importance of the local knowledge, as measured by a country’s transparency. Hence, the coefficient *δ* measures the interaction effect between information transparency and local implementation.

As the control variables are concerned, we mostly rely on the specification by Dreher et al. (2013). As they do, to capture all the economic and political factors that may affect project effectiveness, we include *GDP per capita,* *Population*, *Ethnic Fractionalization* and its squared term (Alesina et al. 2003), *Time in Office* and its squared term (Database of Political Institutions, Beck et al. 2001), *Democracy* (Polity IV, Marshall et al. 2018), and *Government Stability*(ICRG).^38^ Finally, we also control for the size and complexity of the project by including *Total Amount* and *Project Duration.*^39^

Table 7 presents our results. The first six columns of Table 7 show the results from the logistic specification, while columns 7 and 8 present the results of the conditional logit. We start with a parsimonious specification, which only includes project-level variables (column 1). In column 2, we add *Ethnic Fractionalization*, and, in column 3, the full set of control variables.^40^ In columns 4-6 we repeat the previous analysis including the interaction term between a country’s transparency and the indicator for the local implementing agency. In column 7, we add country fixed effects to the specification of column 6. Finally, in column 8 we test for the possibility of non-random assignment of projects. While all these results are reported for comparison, we largely base the discussion on the fully specified model of column 7.

**Table 7:** Project effectiveness

	(1)	(2)	(3)	(4)	(5)	(6)	(7)	(8)
Variables	Logit	Logit	Logit	Logit	Logit	Logit	CLogit	CLogit
Transparency	− 0.279	− 2.081*	− 0.751	− 0.209	− 1.825	− 0.111	3.945*	4.116*
	(0.728)	(1.184)	(1.584)	(0.729)	(1.223)	(1.645)	(2.229)	(2.259)
Local Implementing Agency	0.047	− 0.041	− 0.061	0.751	3.661**	5.356***	4.310*	4.412**
	(0.099)	(0.108)	(0.124)	(0.791)	(1.662)	(1.933)	(2.372)	(2.217)
Local Implementing Agency * Transparency				− 1.005	− 5.095**	− 7.361***	− 6.207**	− 6.347**
				(1.117)	(2.202)	(2.576)	(3.117)	(2.878)
Total amount	0.001*	0.001	0.001*	0.001*	0.001	0.001*	0.000	0.000
	(0.000)	(0.000)	(0.000)	(0.000)	(0.000)	(0.000)	(0.000)	(0.001)
Project duration	− 0.001***	− 0.001***	− 0.001***	− 0.001***	− 0.001***	− 0.001***	− 0.001***	− 0.001***
	(0.000)	(0.000)	(0.000)	(0.000)	(0.000)	(0.000)	(0.000)	(0.000)
Ethnic fractionalization		− 0.006	− 0.019		− 0.005	− 0.018		
		(0.016)	(0.021)		(0.017)	(0.021)		
Ethnic fractionalization, squared		− 0.000	0.000		− 0.000	0.000		
		(0.000)	(0.000)		(0.000)	(0.000)		
Time in office			− 0.036			− 0.038	− 0.079**	− 0.076*
			(0.028)			(0.027)	(0.039)	(0.040)
Time in office, squared			0.001			0.001	0.003***	0.003***
			(0.001)			(0.001)	(0.001)	(0.001)
Democracy			0.022			0.021	0.048	0.048
			(0.018)			(0.018)	(0.040)	(0.039)
Government stability			0.125**			0.127**	0.066	0.077*
			(0.052)			(0.051)	(0.048)	(0.046)
GDP per capita (log)			− 0.302**			− 0.289**	− 5.144***	− 5.180***
			(0.134)			(0.133)	(0.403)	(0.412)
Population (log)			0.015			0.016	− 6.582***	− 6.618***
			(0.045)			(0.045)	(1.592)	(1.552)
								− 0.21
Observations	4,426	2,612	1,991	4,426	2,612	1,991	1,980	1,957
Sector dummies	YES	YES	YES	YES	YES	YES	YES	YES
Regional dummies	YES	YES	YES	YES	YES	YES	NO	NO
Country FE	NO	NO	NO	NO	NO	NO	YES	YES
Year FE	YES	YES	YES	YES	YES	YES	NO	NO

Notes: The dependent variable is a binary indicator Satisfactory, which is equal to 1 if the project is evaluated at least Moderately Satisfactory, and 0 otherwise. Standard errors (clustered at the country level) in parentheses. *** p < 0.01, ** p < 0.05, * p < 0.1

As the control variables are concerned, a project’s success decreases with its complexity (as measured by a project’s duration), with a country’s *GDP per capita* and *Population* (in line with Kilby 2015) and with the duration of a country’s government (*Time in office)* but in a non linear way (as in Dreher et al. 2013). Finally, we find that the project’s amount, democracy and government stability do not seem to be significantly related to a project performance (as in Dreher et al. 2013).

Turning to our variables of interest, we can see that the coefficient of *Transparency* is negative but not significant in the logistic specifications, but turns to be positive and slightly significant with the conditional logit. On the contrary, the interaction between *Transparency* and the indicator for the*Local Implementing Agency,* is always negative. That is, the effect of a local implementing agency on project performance declines as a country’s level of transparency increases.

Finally, following Dreher et al. (2013), column 8 includes the Inverse Mills Ratio (IMR), estimated from the selection stage of a Heckman two-step approach, where the selection stage consists in the the national/local implementation equation presented in Section 5, and the outcome stage is the conditional logistic regression. A significant coefficient of the IMR would indicate that there is selection on unobserved variables, and that excluding it may result in omitted-variable bias. In our case, however, the IMR turns out to be not significant, at conventional levels, whereas the coefficient of the interaction term remains significant.^41^

**Fig. 5:**
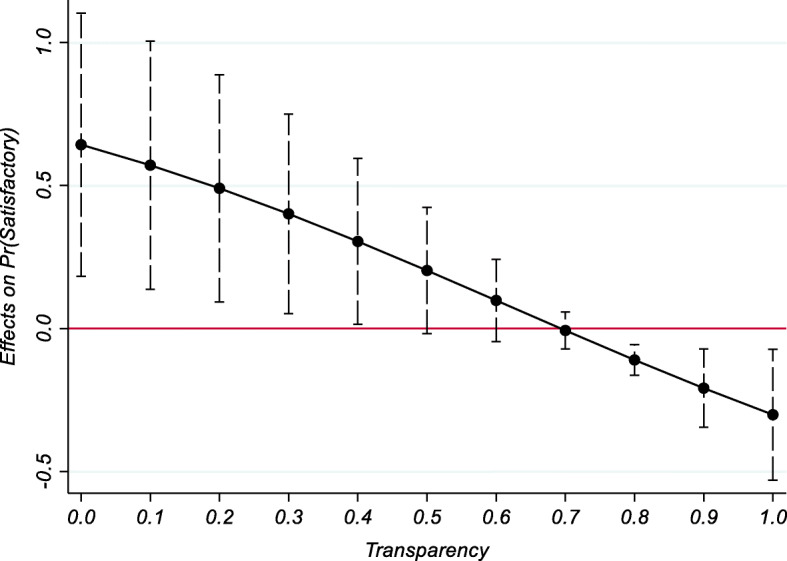
Marginal effects of delegation of implementation

Since we are interested in how the effect of a locally implemented agency changes over the range of the transparency indicator, we calculate average marginal effects using the coefficients from Table 7, column 7. Figures 5 shows them, in tandem with 90-percent confidence intervals. The marginal effect of local implementation on project performance decreases with the intensity of *Transparency*. The effect is significant for low levels of *Transparency*, but turns insignificant at conventional levels when *Transparency* is high. As *Transparency* increases, the recipient’s local knowledge becomes less relevant, so that the comparative advantage of delegating to a local agency becomes increasingly smaller, turning negative for level of *Transparency* greater than a critical value.

Looking at the two extremes, locally implemented projects, in a transparent environment, have a probability of success which is 30 percentage ponts lower than those implemented nationally. On the other hand, choosing a local implementing agency, in a non-transparent setting, increases the probability of a positive evaluation by 64 percentage points. In sum, when the asymmetry of information is more salient and the World Bank chooses a local implementing partner, the informational advantages may overcome the costs due to loss of control. These results complement the analysis by Honig (2019), who finds that tight management control of field agents may have a negative effect, particularly in more unpredictable environments.

## Conclusions

In this paper, we explore the role of information transmission in explaining the choice of a national vs a local level of implementation in World Bank projects. In particular, we empirically assess whether this choice is influenced by the relative importance of the local information at the recipient country level. Exploiting the AidData (2016) dataset that contains information on more than 5800 World Bank projects for the period 1995-2014, we find that, controlling for characteristics at both the country and the project level, (lower) transparency does influence the probability of a project being implemented locally rather than nationally.

Hence, the World Bank is more in need of the recipient’s local knowledge when transparency is lower, consistently with previous results (Marchesi et al. 2011; Dreher et al. 2017, 2018b). More specifically, as transparency increases by one standard deviation, the probability that a project will be implemented locally decreases by three percentage points relative to a baseline of 13 percent local implementation. These results are robust to considering alternative indicators of a country’s transparency. They also hold when restricting the sample to countries with good institutions and to projects that obtained, at least, a “satisfactory” evaluation, which should, at least on average, have less likely been allocated according to geopolitical factors.

What is more, we also show that the effect of a local implementing agency on project performance decreases with a country’s level of transparency. In particular, while a locally implemented project in a non-transparent country has a predicted probability of success which is almost 64 percentage points higher than a nationally implemented one, the probability of success is more than 30 percentage points lower for projects which are locally implemented in a transparent environment. Therefore, when the local knowledge is less relevant and the World Bank still opts for a local implementing partner, the costs due to loss of control may overcome the informational advantages.

Important policy implications arise from these findings. In particular, we identify the transmission of information between government levels, under misaligned interests between them, as an additional element that determines the optimal allocation of project implementation in a country. We suggest that, controlling for project and country characteristics, project implementation should be allocated according to the importance of the local private information and expertise. We should emphasize that the nature of the available data limits our choice to focus on the choice of national versus local project implementation. If new information becomes available, different hypotheses could be made and then tested. For example, we could consider the case in which the World Bank is not restricted to choose between a national or local implementing agency, but would be free to delegate project implementation to different agencies, such as a private agency or an NGO.

Better data allow governments to make better decisions (e.g., Hollyer et al. 2014). Especially in Africa, a paucity of data across swathes of Africa has left governments guessing about where to build schools and roads and “businesses must take shots in the dark when investing in new markets.” (The Economist 2020). What is more, the COVID-19 pandemic further highlights the importance of good *local* health aid in order to access better information about the actual victims of the virus and to fight against it more effectively.

The analysis is, of course, limited in several respects. We do not claim to be able to derive causal relationships, but rather aim to show that the data are in line with our predictions. Our model helps to better explain the existing variations across countries regarding the choice of a national vs local level of implementation and augments the existing literature in an important way. We have shown that the negative coefficient of our variable of interest (Transparency) persists under different specifications. As project effectiveness is concerned, we have shown that our results also hold when we test for the possibility of non-random assignment of projects.

Finally, we would also like to clarify that this paper identifies the transmission of information between government levels, with misaligned interests, as an *additional* mechanism to understand the degree of decentralization in project implementation. We do not claim that informational asymmetry should be taken as the only criterion to explain the choice of implementation level of projects, but we simply argue that it is essential to consider it when discussing reform design and their implementation. For future research, for example, it might be promising to find more reliable proxies for the specific importance of the local information, such as those deriving directly from local sources.

What is more, we should emphasize that political factors (such as the existence of political ties between central and local governments but also political considerations on the World Bank side) would most likely explain the choice of the delegation of implementation power as well. In that respect, a natural extension of the paper would be to explore better the importance of political factors as possible alternative determinants of local implementation. We leave these questions for future research.

## Electronic supplementary material

Below is the link to the electronic supplementary material.

(PDF 538 KB)

## Footnotes

1 Another argument in favor of decentralization is that it improves accountability since citizens can monitor local governments better than central authorities. Bardhan and Mookherjee (2005, 2006), however, demonstrate that accountability, efficiency and equity in service delivery may worsen under decentralization due to the proneness of local governments to pressure from local elites.

2 During the period 1995-2005, over one-quarter of development policy operations listed at least one condition with a decentralization component (Kaiser 2006).

3 In different contexts, Marchesi et al. (2011) have identified and tested the conditions under which it is optimal for the IMF to delegate control to a recipient country in order to maximize the quality of a reform program. More recently, Dreher et al. (2018a) explore the role of information transmission in explaining the optimal degree of decentralization across countries.

4 Greater distance increases costs for communities to organize and demand public goods from the administration, at the same time increasing the costs to the state of supplying public goods and monitoring their quality.

5 Lessmann and Markwardt (2016) find evidence of some heterogeneous effects across the different types of decentralization. While fiscal decentralization negatively affects aid effectiveness, political decentralization does not, and employment decentralization may even have some positive impact.

6 This result is confirmed by Chauvet et al. (2010), who focus on aid effectiveness in a post-conflict environment.

7 More specifically, they find no significant average effect of political influence on the likelihood of a successful project evaluation. However, when examining projects granted to UNSC members experiencing economic vulnerability, they find that project performance suffers.

8 The main simplification is that we assume asymmetry of information to be one-sided, while in all previous related papers (Marchesi et al. 2011; Dreher et al. 2017, 2018b) assume that also the principal owns some non-overlapping private information. Hence, in this simplified version, the model’s information structure follows more closely the original contribution of Crawford and Sobel (1982) and Dessein (2002), as far as only the agent has some private information that is relevant for the implementation of the project, and for the structure of preferences.

9 We posit that the principal cannot commit to an incentive-compatible decision rule in which the Revelation Principle applies (e.g., Dessein 2002). This assumption fits in well with the specific relationship between a central and a local government in which the principal cannot use a standard mechanism to elicit private information from the agent.

10 In a slightly modified framework, the influence of some central governemnt’s interests could be easily included, by allowing a central’s preferred project to be different from *p*^∗^. While this assumption would be more realistic, it would not change the main conclusions of the model.

11 The utility function (1) can be derived from a more general objective function $\widehat {U}^{P}=W(p)$, where *W* is the region’s welfare, which depends on the project *p*. Taking a Taylor expansion of *U*^*P*^(*p*) up to the second term, one obtains the form in (1).

12 The more general function is HCode $\widehat {U}^{A}=W(p)+\theta C(p),$ where *C* are contributions from special interest groups. We assume that *C* decreases with *p* and that the parameter *θ* (0 ≤ *θ* ≤ 1) denotes the importance of lobbies. Using a Taylor expansion of $\widehat {U}^{A}(p)$ up to the second term, one obtains (2).

13 Bordignon et al. (2008), for example, find that when regional lobbies have conflicting interests, lobbying is less damaging for social welfare under centralization than under decentralization.

14 Kilby (2001) considers a principal-agent model as well, to analyze World Bank-funded projects. While he explores the importance of the agency problem by linking World Bank supervision to project performance, we focus on the importance of the local knowledge for choosing the optimal level of implementation.

15 In a broader setting, the World Bank would also likely have its own preferred project $p_{W}^{\ast }\neq p^{\ast }$ For example, the World Bank might care as well for the externalities that the choice of a specific *p* could have on the whole country’s economy, or on the interests of some “special” shareholders.

16 There is substantial empirical evidence linking a country’s geopolitical proximity to the World Bank’s major shareholders with a variety of types of preferential treatment (e.g., Dreher et al. 2009, Kaja and Werker 2010; Kilby 2009, 2013 and Clark and Dolan 2020). For a recent survey, see Dreher and Lang (2019). In the robustness section below, we restrict our sample only to countries with good institutions and to projects obtaining a satisfactory evaluation, which, according to the literature, are less likely to be impacted by political influence (e.g., Dreher et al. 2013, 2018b; Kilby 2015).

17 According to some informal discussion we have conducted with researchers at the Bank, this assumption seems to fit very much what happens in reality. In any case, the World Bank is likely to maintain “control” over the decision of the central government, which is the “true client” of the Bank (the one responsible for repaying the country’s loans). Our theoretical assumptions were then made consistently with this idea.

18 For a discussion on how decisions on aid are negotiated, see Swedlund (2017).

19 We should emphasize here that information transmission is expected to influence the allocation of “implementation power” only for those projects for which it is actually possible to choose (the so called “switchers”). As we will discuss in Section 5.1, there are projects that must be implemented either nationally or locally, and for those no choice can be made.

20 A crucial assumption in our analysis is that the private information of the agent is soft. The motivation for this is that the best thing to do typically depends on a wide range of factors, which the principal may find very hard to foresee, let alone verify their magnitude or nature. Furthermore, even when private information can be certified or verified, this is often a very time-consuming process (Dessein 2002).

21 Hence, in our setting, transparency has a precise meaning, and the two concept of asymmetry of information and transparency are intertwined.

22 On the contrary, we can keep the observation when a project implemented by a private company, or an NGO, is simultaneously implemented also by another (local or national) agency.

23 Indeed, ceteris paribus, the probability of finding a decentralized level of implementation may also depend on the federal structure of the country. In that respect, African countries are generally unitary, while in both Latin America and South Asia there are quite a few large federal countries (think for example of Brazil, Mexico, Argentina, on the one hand, and India, Pakistan and Bangladesh, on the other).

24 Since the same project can involve more than one sector, we decided to consider the sector in which the project is mostly concentrated.

25 For more details on the IEG, see Kilby and Michaelowa (2019).

26 Missing data entries can result from a number of reasons. For example, (i) the recipient government might have the data but does not report them, (ii) the recipient government does not have the information, or (iii) the recipient reports the data to the World Bank but Bank staff choose to not report them, for example because they consider them insufficiently reliable. In all these cases missing data proxy for intransparent environments which, by decreasing the share of “hard” to “soft” information, makes the informational asymmetry between government levels more salient. In turn, when the importance of private “soft” knowledge increases, the principal’s dependency on the local information also increases, with less information being available in cases where no delegation is chosen.

27 More specifically, Hollyer et al. (2014) have found that democracy is more likely to thrive when voters have good information, which in turn boosts foreign direct investment.

28 Press Freedom is highly correlated (0.72) with the Combined Transparency indicator taken from Williams (2015), and significantly correlated (0.4) with the other two Index of transparency (Dreher et al. 2017 and Hollyer et al. 2014).

29 As we show in Table A5, in the Online Appendix, the results do not change excluding budget support from the analysis.

30 As we show in Section 5 below, the role of information is not the same for projects that are more likely to be implemented locally, as compared to those typically dealing with national policies (for instance related to the sector “Public Administration and Finance”).

31 We have included UNSC temporary membership, voting in line with the US in the UNGA, commercial ties with the US or the amount of US aid, but none of those indicators was found to be significantly associated with the decision of a local vs national level of implementation. Results are available on request.

32 Per capita GDP is included in most studies that try to explain decentralization and a country’s (log) population is a proxy for its size that is frequently included in the related literature. See, for example, Panizza (1999) and Treisman (2006).

33 After including country fixed effects, the sign of our variable of interest remains negative, consistently with our main results, albeit its coefficient is not significant. The coefficient of Transparency remains negative but not significant, even after using the updated version of our dataset (in which, incidentally, the number of observations does not increase very much). It becomes significant only after increasing the number of observations thanks to the multiple imputation procedure (i.e., an iterative Markov chain Monte Carlo method to impute missing values, Lall 2016). The results are presented in Table A6, in the Online Appendix.

34 Ordinary logistic analysis in the presence of between-cluster differences may wrongly lead one to conclude that the estimates are negligible, when in fact they may be positive (or stronger) in some clusters and negative (or weaker) in others. Conversely, the multilevel logistic model allows us to disentangle the within-cluster effects from the between-cluster effects.

35 This specification, however, does not include the dummy “federal type” because an updated version of this classification is not available. Since federal type is currently available only up to 2011, keeping it in the specification would have prevented us from significantly increasing the number of observations.

36 As the magnitude of the coefficient of Transparency is concerned, however, while the size of the coefficients obtained from multiple imputation is similar to the one reported in Table 5, the size of the coefficients obtained using the updated dataset is about three times larger. Such big change shows that the updated dataset sample is biased due to missing data being dropped, and, more generally, suggests we should be quite prudent when commenting on the magnitude of these coefficients, despite their sign and significance not being affected.

37 In this specification we include project-level variables only. Results do not change with the full set of control variables. However, since the sample shrinks a lot, and the coefficients of country-level variables are not significant, we prefer to rely on this specification.

38 In our sample, *Time in office* ranges from 1 to 46 years and *Democracy* ranges between -10 (hereditary monarchy) and 10 (consolidated democracy). In particular, about 15 percent of the projects are implemented in autocratic regimes, while 43 percent of them are implemented in democratic countries.

39 As project duration could be endogenous (Kilby 2015), we have also estimated our model by omitting this variable. The results (available on request) remain unchanged.

40 As in Section 5, in order to deal with the high number of missing data, we have replicated the analysis both using the updated version of our dataset and multiple imputation. The results (available on request) show that both sign and significance are similar under these two alternative approaches.

41 We have also tested for the possibility of non-random assignment of projects by including all the control variables used in column 3, Table 5, in the fully specified model presented in this section. The results (available upon request) continue to hold.
