# Supplementary material for: Delegation of implementation in project aid
Source: Rev Int Organ. 2020 Nov 21;16(3):655–87. doi: 10.1007/s11558-020-09396-9 (PMC7679796; doi:10.1007/s11558-020-09396-9)
Supplement: Supplementary file 1 — (PDF 538 KB) [file 11558_2020_9396_MOESM1_ESM.pdf]

Online Appendix

Table A1: Project distribution across countries

Afghanistan	67	Dominica	6	Liberia	30	Senegal	69
Albania	64	Dominican Republic	30	Lithuania	14	Serbia	35
Algeria	17	Ecuador	29	Macedonia, FYR	49	Seychelles	6
Angola	15	Egypt	49	Madagascar	63	Sierra Leone	45
Antigua and Barbuda	1	El Salvador	24	Malawi	56	Slovak Republic	7
Argentina	103	Eritrea	16	Malaysia	4	Slovenia	4
Armenia	79	Estonia	4	Maldives	13	Solomon Islands	15
Azerbaijan	62	Ethiopia	84	Mali	54	South Africa	3
Bangladesh	118	Gabon	5	Marshall Islands	2	South Sudan	5
Barbados	2	Gambia, The	19	Mauritania	37	Sri Lanka	56
Belarus	13	Georgia	70	Mauritius	17	St. Kitts and Nevis	4
Belize	5	Ghana	88	Mexico	97	St. Lucia	15
Benin	47	Grenada	13	Micronesia	3	St. Vincent and the Grenadines	6
Bhutan	18	Guatemala	34	Moldova	55	Swaziland	2
Bolivia	55	Guinea	34	Mongolia	38	Tajikistan	56
Bosnia and Herzegovina	71	Guinea-Bissau	20	Montenegro	18	Tanzania	98
Botswana	3	Guyana	17	Morocco	68	Thailand	18
Brazil	203	Haiti	45	Mozambique	71	Timor-Leste	12
Bulgaria	32	Honduras	60	Myanmar	8	Togo	23
Burkina Faso	60	Hungary	9	Namibia	2	Tonga	13
Burundi	37	India	224	Nepal	56	Trinidad and Tobago	4
Cabo Verde	30	Indonesia	139	Nicaragua	59	Tunisia	46
Cambodia	38	Iran	9	Niger	47	Turkey	71
Cameroon	38	Iraq	7	Nigeria	69	Turkmenistan	2
Central African Republic	14	Jamaica	29	Pakistan	108	Tuvalu	4
Chad	32	Jordan	31	Panama	29	Uganda	71
Chile	20	Kazakhstan	38	Papua New Guinea	20	Ukraine	50
China	233	Kenya	64	Paraguay	19	Uruguay	35
Colombia	86	Kiribati	5	Peru	66	Uzbekistan	29
Comoros	16	Korea, Republic of	4	Philippines	64	Vanuatu	1
Congo, DR	47	Kosovo	22	Poland	30	Venezuela	10
Congo, Rep.	24	Kyrgyz Republic	64	Romania	54	Vietnam	140
Costa Rica	8	Lao PDR	54	Russian Federation	58	Yemen, Republic	76
Cote d'Ivoire	39	Latvia	18	Rwanda	54	Zambia	49
Croatia	49	Lebanon	26	Samoa	16	Zimbabwe	6
Djibouti	28	Lesotho	26	Sao Tome and Principe	12		

Table A2: Variable definitions and sources

Dependent variables	Definition	Source
Local Implementing Agency	Dummy=1 for project implemented by a local agency	Own elaboration from AidData (2016)
Satisfactory	Dummy=1 for projects evaluated at least "Moderately Satisfactory"	Own elaboration from AidData (2016)
**Variables of interest**		
Share of No Missing Data	Share of series included in the World Bank's World Development Indicators for which data are available.	Dreher *et al.* (2017)
Share of No Missing Economic Data	Share of variables related to Economic Policy and Debt included in the World Bank's World Development Indicators for which data are available.	Hollyer *et al.* (2011)
Press Freedom	Status of press freedom: 3 = Free; 2= Partly Free; 1= Not Free.	Freedom House (2012)
Combined Transparency Index	Average of Information Transparency and Accountability Transparency	Williams (2015)
**Project-level control variables**		
Total amount	Commitment Amount (US$, million)	AidData (2016)
Investment project	Dummy=1 for investment projects	AidData (2016)
IBRD	Dummy=1 for IBRD projects	AidData (2016)
Project duration	Number of days between approval and closing dates	Own elaboration from AidData (2016)
**Country-level control variables**		
Ethnic fractionalization	Combined linguistic and racial indicator of fractionalization	Alesina (2003)
Federal system	Dummy=1 for federal government	Norris (2008)
Past local projects	Number of local projects in the country up to the project year	Own elaboration from AidData (2016)
Bureaucratic Quality	Quality of bureaucracy	PRS Group, 2012
Per capita GDP (log)	Log of GDP per capita (con 2000 US$)	World Bank (2013)
Population (log)	Log of total population	World Bank (2013)
Internal conflict	This is an assessment of political violence in the country and its actual or potential impact on governance. A score of 4 points equates to Very Low Risk and a score of 0 points to Very High Risk.	ICRG (2012)
External conflict	The external conflict measure is an assessment both of the risk to the incumbent government from foreign action, ranging from non-violent external pressure (diplomatic pressures, withholding of aid, trade restrictions, territorial disputes, sanctions, etc) to violent external pressure (cross-border conflicts to all-out war). A score of 4 points equates to Very Low Risk and a score of 0 points to Very High Risk.	ICRG (2012)
Time in office	Number of years that the party of the executive has been in office	Beck et al. (2001)
Democracy	Polity2 indicator ranging from -10 (hereditary monarchy) to 10 (consolidated democracy).	Marshall et al. (2014)
Government Stability	Indicator of a maximum of 12 that assess both the government's ability to carry out its declared programs, and its ability to stay in office (with higher value indicating higher instability).	ICRG (2012)

Table A3: Descriptive statistics

	Mean	S.D.	Min	Max
Local implementing agencies	0.13	0.34	0.00	1.00
Share of No Missing data	0.65	0.14	0.04	0.87
Share of No Missing Economic Data	0.89	0.26	0.03	0.98
Press Freedom	1.74	0.64	1	3
Combined transparency Index	50.98	11.59	15.00	76.00
Satisfactory	0.44	0.50	0	1
Past local projects	10.33	27.03	0	171
Total amount	97.68	176.23	0.00	3,750
Investment projects	0.81	0.39	0.00	1.00
IBRD	0.35	0.48	0.00	1.00
Ethnic fractionalization	45.86	22.37	0.20	93.02
Federal system	0.39	0.49	0.00	1.00
Bureaucratic Quality	1.78	0.79	0.00	4.00
GDP per capita (log)	7.01	1.06	4.78	9.58
Population (log)	16.89	1.92	9.19	21.02
Internal conflict	8.79	1.77	0.42	12.00
External conflict	10.02	1.47	2.58	12.00
Time in office	6.72	6.72	1	46
Democracy	2.97	5.81	-10	10
Government Stability	8.49	1.68	3	12

Table A4: Correlations

		(1)	(2)	(3)	(4)	(5)	(6)	(7)	(8)	(9)	(10)	(11)	(12)	(13)	(14)	(15)	(16)
Local Implementing Agencies	(1)	1.00															
Share of No Missing Data	(2)	0.05	1.00														
Share of No Missing Economic Data	(3)	0.08	0.19	1.00													
Press Freedom	(4)	-0.06	0.00	-0.35	1.00												
Combined Transparency Index	(5)	-0.04	0.36	-0.33	0.74	1.00											
Investment projects	(6)	0.16	-0.07	0.06	-0.10	-0.13	1.00										
Total amount	(7)	0.03	0.08	-0.08	0.06	0.06	-0.30	1.00									
IBRD	(8)	0.05	0.21	-0.21	0.16	0.34	-0.03	0.13	1.00								
Ethnic fractionalization	(9)	-0.16	0.04	0.31	0.08	0.02	-0.08	-0.05	-0.28	1.00							
Federal system	(10)	0.39	0.23	-0.04	-0.06	0.03	0.08	0.26	0.30	0.01	1.00						
Past local projects	(11)	0.55	0.18	0.16	-0.13	-0.02	0.12	0.15	0.06	-0.20	0.51	1.00					
Bureaucratic Quality	(12)	0.23	0.00	-0.24	0.31	0.22	0.02	0.22	0.18	-0.12	0.32	0.24	1.00				
GDP per capita (log)	(13)	-0.02	0.21	-0.47	0.38	0.66	-0.10	0.14	0.69	-0.30	0.10	0.01	0.26	1.00			
Population (log)	(14)	0.50	0.14	0.15	-0.19	-0.23	0.13	0.27	0.17	-0.12	0.77	0.68	0.42	-0.14	1.00		
Internal conflict	(15)	0.05	-0.18	-0.23	-0.04	-0.07	0.01	0.02	0.20	-0.43	-0.12	0.00	0.03	0.24	-0.07	1.00	
External conflict	(16)	-0.01	-0.08	-0.14	0.23	0.21	-0.02	0.03	0.32	-0.13	-0.04	-0.05	0.23	0.36	0.00	0.37	1.00

Notes: Simple correlations between all variables included in the empirical section

Table A5: Decentralization of implementing agencies, excluding budget support

	(1)	(2)	(3)	(4)	(5)	(6)
	Logit	Logit	Logit	ML	ML	ML
					Nat. Sec.	Loc. Sec.
Transparency	-2.212***	-1.670***	-4.056**	-4.348***	-8.160***	-3.759***
	(0.338)	(0.486)	(1.987)	(0.598)	(1.329)	(0.627)
Total amount	0.001**	0.000	-0.003***	-0.001***	-0.001	-0.002**
	(0.000)	(0.000)	(0.001)	(0.001)	(0.001)	(0.001)
IBRD	1.015***	0.774***	0.101	0.670*	0.651	0.763**
	(0.131)	(0.169)	(0.349)	(0.358)	(0.637)	(0.355)
Ethnic fractionalization		-0.023***	0.002			
		(0.003)	(0.005)			
Federal system			0.833***			
			(0.278)			
Past local projects			0.033***			
			(0.004)			
Bureaucratic Quality			0.500***			
			(0.184)			
GDP per capita (log)			-0.727***			
			(0.212)			
Population (log)			0.221**			
			(0.092)			
Internal conflict			0.091			
			(0.064)			
External conflict			0.009			
			(0.078)			
Observations	4,076	2,370	2,016	4,076	1,599	2,477
Sector dummies	YES	YES	YES	YES	YES	YES
Regional dummies	YES	YES	YES	YES	YES	YES
Year FE	YES	YES	YES	YES	YES	YES
Number of groups				137	130	135

Notes: Transparency is Share of No Missing Data. Robust standard errors in parentheses. *** p<0.01, ** p<0.05, * p<0.1

Table A6: Country Fixed effects

	(1)	(2)	(3)
VARIABLES	Main sample	Updated dataset	Multiple imputation
Transparency	-0.578	-0.571	-0.770*
	(0.494)	(0.493)	(0.444)
Total amount	-0.002***	-0.002*	-0.002***
	(0.000)	(0.000)	(0.000)
Investment projects	0.006	0.028	0.068
	(0.222)	(0.222)	(0.196)
Bureaucratic Quality	0.178	0.195	0.059
	(0.227)	(0.226)	(0.150)
GDP per capita (log)	0.086	0.116	-0.047
	(0.317)	(0.313)	(0.133)
Population (log)	1.101	1.123	-0.127
	(0.967)	(0.940)	(0.082)
Internal conflict	0.040	0.044	-0.007
	(0.060)	(0.060)	(0.052)
External conflict	-0.096	-0.101	-0.077
	(0.070)	(0.070)	(0.062)
Observations	3114	3134	4049
Number of countries	57	57	70
Sector dummies	YES	YES	YES

Notes: Transparency is Share of No Missing Data. Standard errors in parentheses. *** $p<0.01$, ** $p<0.05$, * $p<0.1$

Table A7: Decentralization of implementing agencies, Logit and Multilevel Logit

	(1)	(2)	(3)	(4)
VARIABLES	Updated dataset		Multiple imputation	
Transparency	-3.856***	-9.202***	-1.382***	-2.783***
	(0.485)	(1.216)	(0.369)	(0.611)
Total amount	0.000	-0.001*	0.000	-0.000**
	(0.000)	(0.000)	(0.000)	(0.000)
Investment projects	0.247	0.095	-0.014	-0.046
	(0.172)	(0.190)	(0.131)	(0.146)
IBRD	1.100***	1.109***	0.764***	0.681***
	(0.132)	(0.248)	(0.123)	(0.167)
Ethnic fractionalization	-0.219	0.761***	-0.020***	-0.020***
	(0.193)	(0.281)	(0.002)	(0.003)
Bureaucratic Quality		0.316***		0.327***
		(0.101)		(0.082)
GDP per capita (log)		-0.285**		-0.239***
		(0.133)		(0.083)
Population (log)		0.453***		0.301***
		(0.054)		(0.033)
Internal conflict		0.082**		-0.049
		(0.041)		(0.037)
External conflict		-0.368***		-0.282***
		(0.047)		(0.042)
Observations	4,566	3,652	5614	5614
Sector dummies	YES	YES	YES	YES
Regional dummies	YES	YES	YES	YES
Year FE	YES	YES	YES	YES

Notes: Transparency is Share of No Missing Data. Robust standard errors in parentheses. *** p<0.01, ** p<0.05, * p<0.1
